# Supplementary material for: aims-PAX: Parallel Active Exploration Enables Expedited Construction of Machine Learning Force Fields for Molecules and Materials
Source: J Chem Inf Model. 2026 Apr 8;66(8):4365–81. doi: 10.1021/acs.jcim.5c02682 (PMC13148546; doi:10.1021/acs.jcim.5c02682)
Supplement: Supplementary file 1 [file ci5c02682_si_001.pdf]

SI: aims-PAX: Parallel Active Exploration Enables Expedited Construction of Machine Learning Force Fields for Molecules and Materials

Tobias Henkes,[1] Shubham Sharma,[2] Alexandre Tkatchenko,[1] Mariana Rossi,[2,3] and Igor Poltavsky*[1]
[1)] *Department of Physics and Materials Science, University of Luxembourg, L-1511 Luxembourg, Luxembourg*
[2)] *Max Planck Institute for the Structure and Dynamics of Matter, 22761 Hamburg, Germany*
[3)] *Yusuf Hamied Department of Chemistry, Lensfield Road, Cambridge CB21EW, UK*

(*Electronic mail: igor.poltavskyi@uni.lu)

SUPPLEMENTARY INFORMATION

S1. PARALLEL COMPUTATION WITH ASI AND PARSL DURING IDG AND AL

During both initial dataset generation (IDG) and active learning (AL) aims-PAX enables parallel computation of reference calculations. In the following subsections we provide technical information on this features and highlight their advantages.

A. During Initial Dataset Generation

In the IDG, PARSL[1] can be applied, similar to the approach used in PSIFLOW[2]. PARSL enables the automatic orchestration of parallel computations on HPC clusters, allowing users to perform DFT calculations on sampled geometries across multiple nodes simultaneously. This is particularly advantageous when a general-purpose model is used to sample geometries via MD during the IDG. Since the reference calculations for these configurations are entirely independent, the use of PARSL across multiple nodes enables drastic acceleration of the workflow.

B. During Active Learning

Due to the multi-trajectory strategy, MLFF sampling can continue on other trajectories even when one is halted for exceeding the uncertainty threshold. While active trajectories are propagated on the GPU, reference calculations for high-uncertainty geometries can be performed independently on a CPU. In aims-PAX, we implemented two distinct approaches for this task.

In the first approach, calculations are performed using FHI-aims[3] compiled as a library and interfaced through the ATOMIC SIMULATION INTERFACE[4]. This allows an instance of FHI-aims to run continuously, eliminating the need to reinitialize the DFT code for every calculation. Removing this overhead is particularly valuable for smaller systems. Additionally, by leveraging MPI, the DFT calculation can run as a separate process from the MLFF inference.

In the second approach, similar to the IDG, PARSL[1] is used to distribute DFT calculations across multiple nodes in parallel. While the MLFF propagates the remaining trajectories, PARSL automatically submits new jobs for reference data and handles the subsequent data retrieval.

Furthermore, the number of DFT workers can be adapted dynamically up to a user-defined maximum. This flexible resource allocation ensures that workers are neither idle nor overloaded, which is especially useful in AL where the demand for new data fluctuates. For instance, the workflow may encounter long sequences where trajectories remain certain, followed by intervals where multiple trajectories simultaneously reach high-uncertainty regions. Due to these computational advantages, the PARSL-based approach is designated as the default procedure in aims-PAX.

S2. EFFECT OF ENSEMBLE SIZE ON THE UNCERTAINTY MEASURE

In order to investigate the effect of increasing the size of the model ensemble on the reliability of the uncertainty measure two more aims-PAX runs were performed with 8 and 16 models, respectively. The workflow parameters were otherwise kept the same as described in Section V. Just as done with 4 ensemble members, reference calculations were done at regular intervals and compared to the computed uncertainty. The results of these calculations are summarized in Fig. S1.

In the first row of Fig. S1, the correlation between error and uncertainty for the total AL runs is shown. The Pearson correlation coefficient is 0.89 and 0.83 for 8 and 16 ensemble members, respectively. In comparison, for 4 members, the coefficient was 0.80 (see Fig. 2b). Based on these findings, there is no significant improvement when increasing the number of ensemble members for our use case.

In the second row of Fig. S1, the correlation between error and uncertainty is shown for sections of the aims-PAX run for the individual trajectories. In the case of 8 ensemble members, the correlation for two of the trajectories (orange, green) is slowly decreasing while one (blue) is first decreasing and then rising again for the last section. In contrast, with 16 members the correlation of all three trajectories is decreasing over the course of the AL run. In comparison, for the run with 4 members the correlation also decreased and became negative for one of the trajectories. For the other two trajectories the correlation became more positive again (see Fig. 2c). Despite these individual differences in behavior, there is no clear, advantageous trend that would warrant the considerable increase of computational cost associated with using the larger model ensembles.

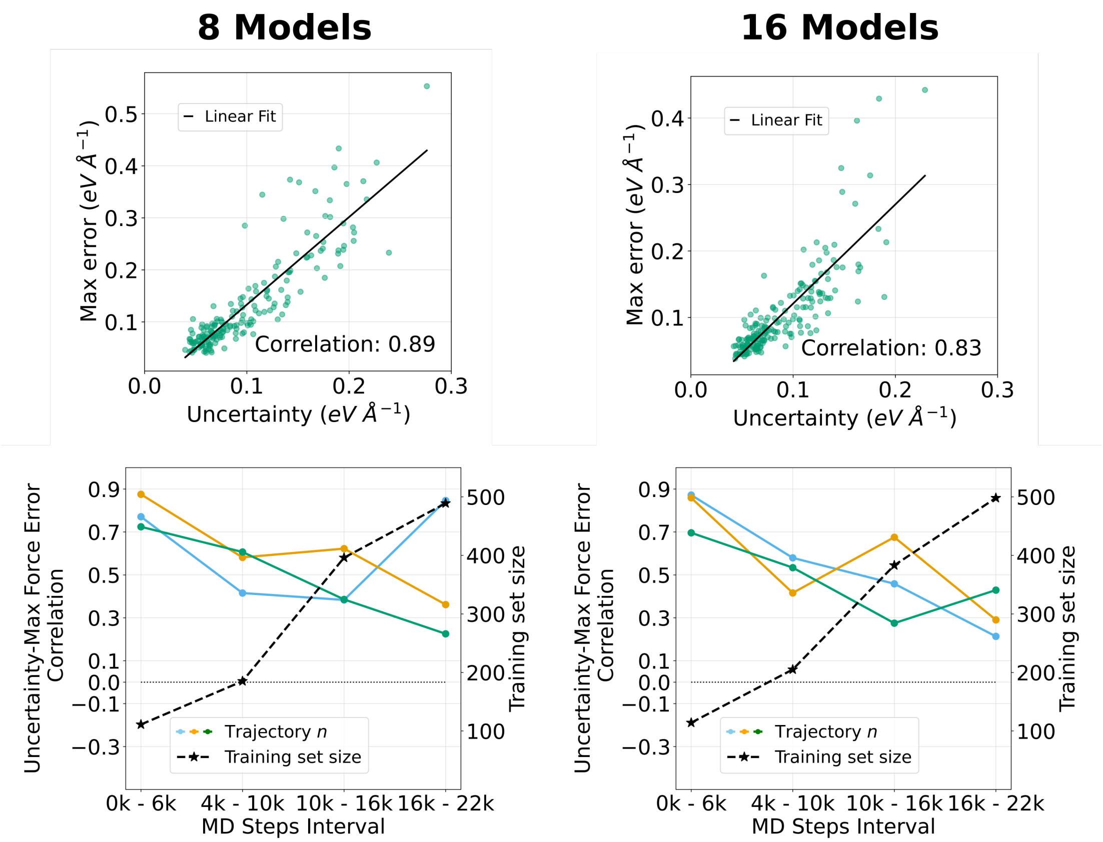

Figure S1: Correlation between uncertainty and actual error for ensembles for two aims-PAX runs with 8 and 16 members: (First row) Actual maximum force error vs. model uncertainty with Pearson correlation coefficient over the whole AL workflow. A linear fit is shown as a guide to the eye. (Second Row) Model uncertainty, actual maximum force error, uncertainty threshold and training set size as a function of MD steps throughout the AL procedure.

S3. ACCURACY AND STABILITY OF THE MODELS FOR AC-F-A5-K

The average MAEs for different numbers of concurrent trajectories during the AL procedure are reported along with their variances in Table S I. At 300 K, all models achieved MAEs between 16 and 18 meV/Å, independent of the number of trajectories used. Similarly, for the 500 and 700 K test sets, MAEs ranged from 27 to 30 and 37 to 40 meV/Å, respectively; showing negligible dependence on the number of parallel trajectories.

	**MAE Forces (meV/Å)**		
**# Traj.**	300 K	500 K	700 K
1	17.93 ± 2.28	27.07 ± 0.25	37.73 ± 0.84
4	16.60 ± 0.44	28.50 ± 0.61	37.47 ± 1.11
8	16.43 ± 0.67	28.73 ± 0.99	37.93 ± 1.36
16	17.20 ± 0.82	29.67 ± 0.95	39.70 ± 1.77
32	16.83 ± 0.38	29.77 ± 1.30	38.73 ± 1.36

Table S I: Mean absolute test errors of models of Ac-F-A5-K acquired using aims-PAX: Models were created from various number of sampling trajectories (# Traj.). Average and standard deviation over the best models from three separate aims-PAX runs.

The results of all MD stability tests are summarized in Table S II. At 300 K, all MD trajectories remained stable, regardless of the number of trajectories used during the AL process. At 500 K, the temperature used during AL, nearly all MD runs were also stable, with only one exception: a single unstable simulation was observed for the model trained using eight parallel AL trajectories. In contrast, at the elevated temperature of 700 K, instability was observed in at least one MD run for every MLFF model tested. Specifically, for models trained with 8 and 32 trajectories, 7 out of 12 MD simulations were unstable. For the model obtained from a single-trajectory AL run, 3 simulations were unstable. Finally, for models trained with 4 and 16 trajectories, 1 and 2 simulations were unstable, respectively. These results align with the sampling strategy used in AL: since the training data were collected at 500 K, it is expected that MD simulations at or below this temperature (e.g., 300 K and 500 K) remain stable, as the MLFF is unlikely to encounter configurations outside its training domain.

At 700 K, however, the MD trajectories explore more diverse and potentially unseen regions of configuration space, which can lead to instability due to extrapolation beyond the model's training domain.

	# Stable MD Runs		
**# Traj.**	300 K	500 K	700 K
1	12	12	9
4	12	12	11
8	12	11	7
16	12	12	10
32	12	12	7

Table S II: Number of stable MD simulations performed with models of Ac-F-A5-K acquired using aims-PAX: Various number of trajectories (# Traj.) were used for sampling at multiple temperatures. Three models were obtained from separate aims-PAX runs and four simulations were run for each, thus a maximum of 12 stable MD runs can be achieved per category. Stability was defined as no bonded atoms separated by more than 2 Å.

S4. TRAINING MODELS FROM SCRATCH

Temperature	MAE Forces (meV/Å)	# Stable MD Runs
300 K	17.53 ± 0.90	12
500 K	28.03 ± 1.03	12
700 K	37.77 ± 1.25	10

Table S III: Stability and test errors for models of Ac-F-A5-K trained from scratch: Mean absolute test errors (mean ± standard deviation) and number of stable MD simulations at various temperatures for models trained from scratch on data acquired via an aims-PAX run. MAE values are averaged over three models with different seeds. Each model was used to generate 4 MD simulations, for a total of 12 per temperature. Stability was defined as no bonded atoms separated by more than 2 Å.

In order to assess the difference between continuously training the models during AL and training models from scratch afterwards, we trained 3 models with different seeds on the dataset of Ac-F-A5-K, acquired through the aims-PAX run with 4 trajectories as described in Section IV. All settings for training, testing, MD simulations and the model architecture were kept the same. The results for the accuracy and stability are shown in Table S III. Comparing with results in Tables SI and SII, there is no meaningful difference between training from scratch and using continual learning (CL) observable for accuracy and stability. Given that CL is computational more efficient, it is the mode of action used in aims-PAX.

S5. ACCURACY OF THE MODELS FOR MD17

The test errors of the MLFFs trained using aims-PAX and a manual, "traditional" approach are shown in Fig. S2. The dashed line shows the MAE on the forces across all species, and the bars show system-specific MAEs. Both the model trained from scratch and the model generated using aims-PAX perform similarly with an overall MAE of 22.6 and 22.9 meV/Å, respectively. In particular, for benzene and naphthalene, both models achieved low errors of 5.7 and 4.5 meV/Å, as well as 16.4 and 13.8 meV/Å, for aims-PAX and the model traditionally trained, respectively.

The model from the AL procedure is only slightly less accurate, while trained on only 33 and 39 points for benzene and naphthalene, respectively, compared to 100 points each for the reference model. Similarly, the model from aims-PAX was trained on only 39 toluene geometries and achieved an error of 22.1 meV/Å, while the traditional model was trained on 100 geometries and achieved an error of 13.8 meV/Å.

Both model types exhibit their largest errors on aspirin and malonaldehyde, with MAEs of 32.7 and 35.1 meV/Å, respectively, for the model created manually and 28.1 meV/Å for aspirin and 33.0 meV/Å for malonaldehyde for the model obtained *via* aims-PAX.

S6. DIFFUSION COEFFICIENTS OF BULK WATER AND SOLVATED PARACETAMOL

To characterize the mobility of water molecules in different environments, the self-diffusion coefficient (D) was calculated using the Mean Squared Displacement (MSD) derived from molecular dynamics (MD) trajectories. To isolate the intrinsic motion of solvent molecules from global system translations, relative coordinates were established for each frame. In the bulk case, the total center of mass (COM) of the simulation cell was used as the reference point. Conversely, for the analysis of the first solvation shell, coordinates were transformed into the local reference frame of the solute.

For the shell-specific analysis, water molecules were dynamically assigned to the first solvation shell based on a distance cutoff (r_{cut}) of 5.0 Å. Oxygen atoms located within this radius of the solute COM at the initial frame (t_0) of a specific calculation window were tracked for the duration of that window. A sliding window approach was employed, utilizing a window length of 10.0 ps with new windows initiated every 5 frames. The MSD was calculated for oxygen atoms according to the following expression:

$$\mathrm{MSD}(\tau) = \langle |\mathbf{r}_i(t_0+\tau) - \mathbf{r}_i(t_0)|^2 \rangle \tag{1}$$

In this formulation, $\mathbf{r}_i$ represents the relative position of the i-th oxygen atom, t_0 is the start time of the window, and τ is the lag time. For the shell case, the ensemble average $\langle \dots \rangle$ was restricted specifically to those molecules satisfying the r_{cut} criterion at the window's origin, t_0.

The diffusion coefficient was ultimately extracted by fitting the linear regime of the MSD curve, defined as the latter 50%

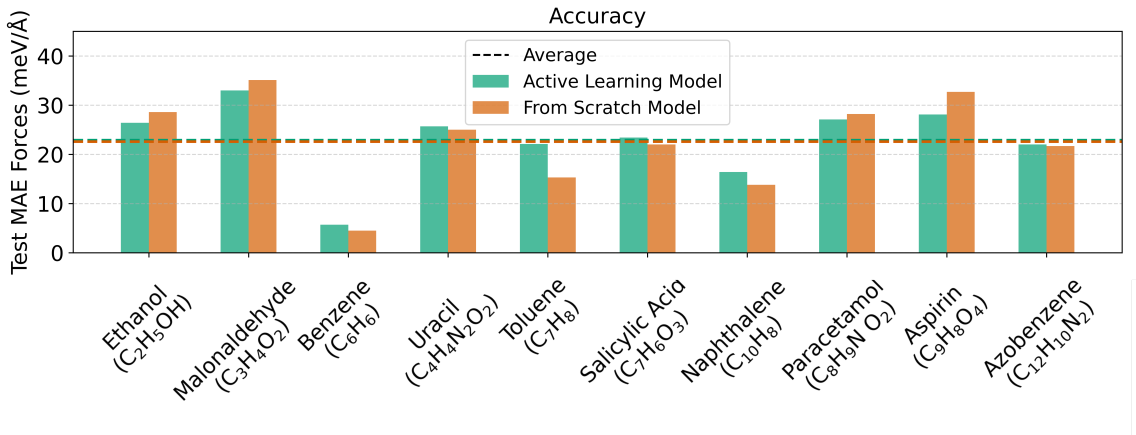

Figure S2: Accuracy of MLFFs acquired using aims-PAX and a model trained from a manually created dataset for MD17: The dashed line indicates average performance across all systems. Systems are sorted from the smallest to the largest number of atoms in the molecule

of the window duration ranging from 5.0 to 10.0 ps, to the Einstein relation:

$$D = \lim_{\tau \to \infty} \frac{1}{6\tau} \mathrm{MSD}(\tau) \quad (2)$$

The slope of this linear fit, where $\mathrm{MSD} = 6D\tau + C$, provided the diffusion coefficient in units of Å^2/ps. A summary of the specific parameters used for both the bulk and shell analysis modes is provided in Table S IV.

Parameter	Bulk	Shell
Time Step (Δt)	0.1 ps	0.001 ps
Window Length	10.0 ps	10.0 ps
Reference Frame	System COM	Solute COM
Selection Criterion	All Oxygen atoms	$d \leq 5.0$ Å from solute

Table S IV: Summary of trajectory analysis parameters for bulk and shell diffusion: Comparison of the temporal and spatial constraints applied during the MSD calculation. The time step and reference frame were adjusted per mode to ensure accurate tracking of solvent mobility relative to the appropriate center of mass.

We performed this analysis for four 1.2 ns long bulk water simulations (NPT, 300 K, 0.5 fs timestep, see Table S X and S XI for thermo- and barostat settings) and the 36 800 ps long solvated paracetamol simulations (see Section V) used in this work.

For the bulk case the average diffusion is (0.0023 ± 0.0003) Å^2/ps and for the shell the value is (0.0019 ± 0.0009) Å^2/ps.

Firstly, the diffusion coefficient for bulk water is one order of magnitude lower than that acquired from a *ab initio* MD simulation of water using the underlying PBE functional. Namely, the latter results in diffusion coefficients of 0.018 Å^2/ps.[5] Secondly, the shell diffusion shows a large standard deviation of ± 0.0009 Å^2/ps or roughly 47% in relative terms.

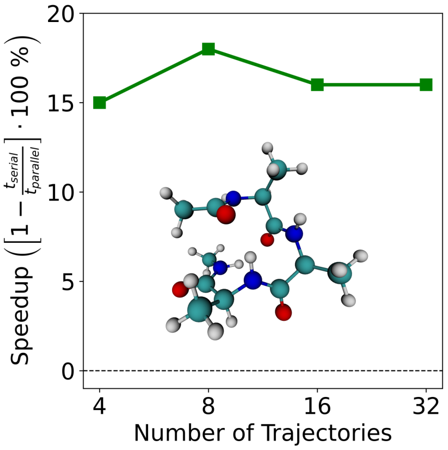

Figure S3: Speedup of the parallel version over the serial version: Application to Ac-Ala3-NHMe running on a single CPU node with 128 cores and 1 GPU card.

In conclusion, the acquired model strongly overestimates diffusion barriers which leads to very low diffusion coefficients and hindrance of convergence in the case of the solvent shell diffusivity. To overcome this challenge, a more accurate underlying density functional[6] alongside more exhaustive sampling during AL and inference is needed.

S7. SPEEDUP OF CPU/GPU PARALLEL aims-PAX VERSION

The results regarding the speedup using the CPU/GPU parallel aims-PAX version are summarized in Fig. S3. We found that using 4 or more trajectories results in a speedup of 15 %. The largest speed-up can be observed with 8 trajectories with 18 %. For 16 and 32 the speedup is slightly lower than for

8 trajectories at 16 % for both runs. The speed-up observed when enabling more trajectories is caused by DFT calculations that run concurrently with the sampling of new points and training. Furthermore, a slight speed-up is observed between 4 and 8 trajectories, likely due to the reduced probability of no trajectory being propagated at a given time. Because trajectories are stopped when the uncertainty threshold is exceeded, it is more likely that all trajectories will stop if there are fewer of them, thus halting all AL progress.

The same DFT settings used for Ac-F-A5-K were used for Ac-A3-NHMe during AL. The only exception is that no dispersion correction was applied. For generating the initial data set using aims-PAX the same settings as for Ac-F-A5-K were used.

For the AL workflow with aims-PAX, the same settings were used as for Ac-F-A5-K, except that the parallel aims-PAX version was used and the procedure stopped when the training set size of 200 was reached. Also, the same MACE architecture as described in Table I under *Ac-F-A5-K (small)* was used.

S8. NUMBER OF MD STEPS PER TRAJECTORY

To give an overview of how many MD steps were performed during a typical aims-PAX run, we provide said information for each of the applications shown in this work in Tables S V, S VI, S VII and S VIII.

S9. COMPUTATIONAL DETAILS: IMPORTANT PARAMETERS

In Table S IX we provide important parameters regarding the various aims-PAX runs performed in this work.

In Tables S X and S XI settings for thermostats and barostats are listed.

S10. TECHNICAL DETAILS OF aims-PAX

Here we provide some technical details of the inner workings of aims-PAX. We focus on the training during AL, explain how data as well as failed SCF convergence are handled and highlight slight differences between the order of operations in the serial and parallel AL algorithm.

A. Training during Initial Dataset Generation and Active Learning

During the initial dataset generation, the user decides how many points are selected for each ensemble member during each sampling step. For example, the user specifies that 5 structures per member are to be selected. aims-PAX then runs the sampling algorithm, and once 5 points are picked from the trajectory for each member (and labels are computed), the models are trained for a user specified number of epochs, namely `intermediate_epochs`. This process is repeated multiple times *i.e.* running sampling, picking points, labeling and training. The rationale behind this is, that if the user wants to have a specific accuracy of the models before running AL, aims-PAX makes sure that not too many structures are sampled and DFT calculations are performed.

During the AL procedure each trajectory is associated with a state. Technically speaking, a loop is performed over all trajectories and depending on their state, different actions are performed. At the beginning of AL, all trajectories have the state `running`, which means the sampling algorithm is performed. Once a point is picked for labeling, the state of this specific trajectory is set to `waiting` until the DFT calculation is done and the results were received. This then changes the state to `training` and the user specified number of training epochs are performed, these are called `intermediate_epochs_al`. Afterwards, aims-PAX continues the loop over the trajectories. Only once a maximum, user-specified number of epochs, `max_epochs_worker`, is reached, the trajectory's state swtiches back to `running`. This is done to enable other trajectories to continue sampling, potentially triggering new DFT calculations, which can then run while the models are trained. In addition, this means that the trajectories are always propagated with continuously updated model parameters.

B. Resetting the Optimizer during Active Learning

While training during AL, the weights are not reset when a new point is added. We have seen that repeatedly using the updated weights can result in the model being stuck in a minimum. We found it advantageous in this case to reset the optimizer state if the model is not improved after `max_epochs_worker` epochs (see Section S10 A). This deletes the history of the adaptive optimizer (e.g. Adam or AMSGrad), resulting in a larger learning rate which helps the model to leave the local minimum.

C. Handling of new data points during Active Learning

Once a new point is selected and labeled during AL, it is either added in the training or validation set. To which dataset the new point contributes depends on a user specified ratio, that is kept consistent, e.g. 0.5, which means points are added to both sets alternately. In contrast to the IDG, both datasets are shared across models (except for the initial starting points that are present before the AL).

D. Handling of Failed SCF Convergence

During the IDG, if a DFT computation does not converge the geometry is discarded from the dataset. The procedure then just continues until any stopping criterion is met. However, we have not noticed any instances where SCF convergence could not be achieved for a geometry generated by the

Trajectory Idx.	MD steps
0	24420
1	24340
2	24400

Table S V: Number of MD steps per trajectory that was reached during an aims-PAX run of the peptide Ac-F-A5-K.

Trajectory Idx.	MD steps
0 (Aspirin)	80800
1 (Azobenzene)	81925
2 (Benzene)	83400
3 (Ethanol)	81825
4 (Malonaldehyde)	81250
5 (Naphtalene)	83375
6 (Paracetamol)	82150
7 (Salicylic Acid)	82275
8 (Toluene)	83350
9 (Uracil)	81850

Table S VI: Number of MD steps per trajectory that was reached during an aims-PAX run of the molecules in the MD17 dataset

GP model for our systems.
In the case of AL, points where the SCF cycles do not converge are also discarded. On the trajectory where this is the case, a checkpoint geometry is loaded. This checkpoint is updated each time a selected structure is successfully labeled using DFT and the data is added to the training set. This ensures, that if the checkpoint is loaded, the MD continues from a geometry that is known to the MLFF.

E. Operational Differences: Serial vs. Parallel Version

While the overall AL workflow of aims-PAX is the same for its serial and parallel versions, there are slight differences that we want to point out. In the case of the serial procedure, the sampling and training of the ML models is halted if DFT calculations are performed. Afterwards, the model parameters are updated on the new data. Practically this results in all trajectories being propagated with the new information. For the parallel version, other trajectories can be propagated during the DFT calculation, meaning that sampling is done without the information of the current DFT calculation. While it can mean that potentially redundant points are sampled, the computational benefit, and thus possibility of scaling up the workflow, outweighs this inefficiency.

REFERENCES

[1] Y. Babuji, A. Woodard, Z. Li, D. S. Katz, B. Clifford, R. Kumar, L. Lacinski, R. Chard, J. Wozniak, I. Foster, M. Wilde, and K. Chard, "Parsl: Pervasive parallel programming in python," in *28th ACM International Symposium on High-Performance Parallel and Distributed Computing (HPDC)* (2019).

[2] S. Vandenhaute, M. Cools-Ceuppens, S. DeKeyser, T. Verstraelen, and V. Van Speybroeck, "Machine learning potentials for metal-organic frameworks using an incremental learning approach," npj Computational Materials **9** (2023), 10.1038/s41524-023-00969-x.

[3] J. W. Abbott, C. M. Acosta, A. Akkoush, A. Ambrosetti, V. Atalla, A. Bagrets, J. Behler, D. Berger, B. Bieniek, J. Björk, V. Blum, S. Bohloul, C. L. Box, N. Boyer, D. S. Brambila, G. A. Bramley, K. R. Bryenton, M. Camarasa-Gómez, C. Carbogno, F. Caruso, S. Chutia, M. Ceriotti, G. Csányi, W. Dawson, F. A. Delesma, F. D. Sala, B. Delley, R. A. D. Jr., M. Dragoumi, S. Driessen, M. Dvorak, S. Erker, F. Evers, E. Fabiano, M. R. Farrow, F. Fiebig, J. Filser, L. Foppa, L. Gallandi, A. Garcia, R. Gehrke, S. Ghan, L. M. Ghiringhelli, M. Glass, S. Goedecker, D. Golze, M. Gramzow, J. A. Green, A. Grisafi, A. Grüneis, J. Günzl, S. Gutzeit, S. J. Hall, F. Hanke, V. Havu, X. He, J. Hekele, O. Hellman, U. Herath, J. Hermann, D. Hernangómez-Pérez, O. T. Hofmann, J. Hoja, S. Hollweger, L. Hörmann, B. Hourahine, W. B. How, W. P. Huhn, M. Hülsberg, T. Jacob, S. P. Jand, H. Jiang, E. R. Johnson, W. Jürgens, J. M. Kahk, Y. Kanai, K. Kang, P. Karpov, E. Keller, R. Kempt, D. Khan, M. Kick, B. P. Klein, J. Kloppenburg, A. Knoll, F. Knoop, F. Knuth, S. S. Köcher, J. Kockläuner, S. Kokott, T. Körzdörfer, H.-H. Kowalski, P. Kratzer, P. Kůs, R. Laasner, B. Lang, B. Lange, M. F. Langer, A. H. Larsen, H. Lederer, S. Lehtola, M.-O. Lenz-Himmer, M. Leucke, S. Levchenko, A. Lewis, O. A. von Lilienfeld, K. Lion, W. Lipsunen, J. Lischner, Y. Litman, C. Liu, Q.-L. Liu, A. J. Logsdail, M. Lorke, Z. Lou, I. Mandzhieva, A. Marek, J. T. Margraf, R. J. Maurer, T. Melson, F. Merz, J. Meyer, G. S. Michelitsch, T. Mizoguchi, E. Moerman, D. Morgan, J. Morgenstein, J. Moussa, A. S. Nair, L. Nemec, H. Oberhofer, A. O. de-la Roza, R. L. Panadés-Barrueta, T. Patlolla, M. Pogodaeva, A. Pöppl, A. J. A. Price, T. A. R. Purcell, J. Quan, N. Raimbault, M. Rampp, K. Rasim, R. Redmer, X. Ren, K. Reuter, N. A. Richter, S. Ringe, P. Rinke, S. P. Rittmeyer, H. I. Rivera-Arrieta, M. Ropo, M. Rossi, V. Ruiz, N. Rybin, A. Sanfilippo, M. Scheffler, C. Scheurer, C. Schober, F. Schubert, T. Shen, C. Shepard, H. Shang, K. Shibata, A. Sobolev, R. Song, A. Soon, D. T. Speckhard, P. V. Stishenko, M. Tahir, I. Takahara, J. Tang, Z. Tang, T. Theis, F. Theiss, A. Tkatchenko, M. Todorović, G. Trenins, O. T. Unke, Álvaro Vázquez-Mayagoitia, O. van Vuren, D. Waldschmidt, H. Wang, Y. Wang, J. Wieferink, J. Wilhelm, S. Woodley, J. Xu, Y. Xu, Y. Yao, Y. Yao, M. Yoon, V. W. zhe Yu, Z. Yuan, M. Zacharias, I. Y. Zhang, M.-Y. Zhang, W. Zhang, R. Zhao, S. Zhao, R. Zhou, Y. Zhou, and T. Zhu, "Roadmap on advancements of the fhi-aims software package," (2025), arXiv:2505.00125 [cond-mat.mtrl-sci].

[4] P. V. Stishenko, T. W. Keal, S. M. Woodley, V. Blum, B. Hourahine, R. J. Maurer, and A. J. Logsdail, "Atomic simulation interface (asi): application programming interface for electronic structure codes," Journal of Open Source Software **8**, 5186 (2023).

[5] T. A. Pham, T. Ogitsu, E. Y. Lau, and E. Schwegler, "Structure and dynamics of aqueous solutions from pbe-based first-principles molecular dynamics simulations," The Journal of Chemical Physics **145** (2016), 10.1063/1.4964865.

[6] J. Villard, M. P. Bircher, and U. Rothlisberger, "Structure and dynamics of liquid water from ab initio simulations: adding minnesota density functionals to jacob's ladder," Chemical Science **15**, 4434–4451 (2024).

**Trajectory Idx.**	**MD steps**
0 (Paracetamol in gas phase)	20400
1 (Paracetamol in water cluster, 300 K)	29100
2 (Paracetamol in water cluster, 350 K)	33300
3 (Paracetamol in water cluster, 400 K)	32200
4 (Bulk water, 300 K)	54950
5 (Bulk water, 400 K)	48150
6 (Bulk water, 500 K)	39600

Table S VII: Number of MD steps per trajectory that was reached during an aims-PAX run for creating a MLFF of solvated Paracetamol.

**Trajectory Idx.**	**MD steps**	**Trajectory Idx.**	**MD steps**	**Trajectory Idx.**	**MD steps**	**Trajectory Idx.**	**MD steps**
0	390	8	300	16	410	24	2750
1	3650	9	3990	17	1180	25	2150
2	1370	10	180	18	540	26	660
3	4670	11	1660	19	790	27	500
4	390	12	1150	20	530	28	600
5	1400	13	370	21	1270	29	320
6	680	14	920	22	1250	30	4440
7	3760	15	5190	23	260	31	510

Table S VIII: MD steps per trajectory for $CsPbI_3$.

**Parameter**	**Ac-F-A5-K**	**MD17**	**Paracetamol+H_2O**	**$CsPbI_3$**
Stat. Ensemble	NVT	NVT	NVT & NPT	NPT
Timestep (fs)	1	1	1 & 0.5	1
Parallel trajectories	3	10	7	32
Uncertainty checks every ... steps	20	25	50	10
Uncertainty window size	400	400	400	400
Threshold Scaling parameters	0.	0.	0.	0.2
# train. points sampled	500	1000	1000	100
Threshold frozen (# train. points)	No	500	500	No

Table S IX: Key parameters for aims-PAX runs and MD simulations therein applied to various systems throughout the study.

**Parameter**	**Ac-F-A5-K**	**MD17**	**Paracetamol+H_2O**	**$CsPbI_3$**
Thermostat	Langevin	Langevin	Langevin & Nosé-Hoover	Melchionna (NH)
T (K)	500	500	300-600	300
Friction coefficient (fs^{-1})	0.001	0.001	0.001	-
Time scale factor ($Å\sqrt{u/eV}$)	-	-	100	30
# chains	-	-	3	1
# sub-steps	-	-	1	1

Table S X: Thermostat settings applied in the MD simulations used in aims-PAX runs for various systems throughout the study.

**Parameter**	**Paracetamol+H_2O**	**$CsPbI_3$**
Barostat	Martyna–Tobias–Klein	Parrinello-Rahman
P (atm)	1	1
Cell Thermostat	Nosé-Hoover Chain	Melchionna (NH)
Cell # chains	3	1
Time-scale factor ($Å\sqrt{u/eV}$)	1000	100

Table S XI: Barostat settings updated with cell thermostatting details.
